# *SlIAA9* Mutation Maintains Photosynthetic Capabilities under Heat-Stress Conditions

**DOI:** 10.3390/plants12020378

**Published:** 2023-01-13

**Authors:** Bayu Pradana Nur Rahmat, Grace Octavianis, Rahmat Budiarto, Nurul Jadid, Ani Widiastuti, Deden Derajat Matra, Hiroshi Ezura, Syariful Mubarok

**Affiliations:** 1 Master Program of Agronomy, Faculty of Agriculture, Universitas Padjadjaran, Sumedang 45363, Indonesia; 2 Under Graduate Program of Agrotechnology, Faculty of Agriculture, Universitas Padjadjaran, Sumedang 45363, Indonesia; 3 Department of Agronomy, Faculty of Agriculture, Universitas Padjadjaran, Sumedang 45363, Indonesia; 4 Department of Biology, Institut Teknologi Sepuluh Nopember, Surabaya 60111, Indonesia; 5 Department of Plant Protection, Faculty of Agriculture, Universitas Gadjah Mada, Yogyakarta 55281, Indonesia; 6 Department of Agronomy and Horticulture, Faculty of Agriculture, IPB University, Bogor 16680, Indonesia; 7 Faculty of Life and Environmental Sciences, University of Tsukuba, Tsukuba 305-8577, Japan; 8 Tsukuba Plant Innovation Research Center, University of Tsukuba, Tsukuba 305-8577, Japan

**Keywords:** tomato, heat stress, heat tolerance, *SlIAA9* mutation, photosynthetic rate, photosynthesis

## Abstract

Tomato is one of the most widely consumed horticultural products. However, tomato is very sensitive to changes in temperature. Daily average temperatures above 32 °C severely reduced tomato plant growth, development, and productivity. Therefore, climate change-induced global warming is a major threat to future tomato production. Good photosynthetic capability under heat stress conditions is known to be a major sign of heat tolerance. Tomato *INDOLE-ACETIC-ACID (SlIAA9*) is a transcriptional repressor in auxin signaling. *SlIAA9* mutation caused heightened endogenous auxin response and biosynthesis within plant tissues. In this study, we studied the photosynthetic capability of *iaa9-3* and *iaa9-5* mutants under heat-stress conditions. We discovered that both *iaa9-3* and *iaa9-5* could maintain their photosynthetic capability after 14 days of heat treatment (>40 °C), differing from Wild Type-Micro-Tom (WT-MT) tomato. Both *iaa9* mutants had higher net photosynthetic rate, stomatal conductance, leaf total chlorophyll, leaf carotenoids, Fv/Fm value, and lower leaf MDA than WT-MT. These results suggested that the *SlIAA9* mutation benefits plant adaptation to heat stress.

## 1. Introduction

Tomato (*Solanum lycopersicum*) is one of the most important horticultural crops grown worldwide, reflected by its prevalence in our culinary culture [1], and its economic importance [2]. The annual consumption of tomato fruit worldwide reached 180 million tons [3]. In the future, one of the most pressing concerns regarding the future of tomato fruit production is climate change. Climate change increases air and soil temperature, greatly affecting tomato productivity and other temperature-sensitive crops [4,5]. Day temperatures exceeding 26 °C and night temperatures exceeding 20 °C significantly decrease tomato yield [6], due to pollen infertility [7], flower abscission [8], reduced fruit set [7], and disrupted photosynthesis process [9].

Photosynthesis is known to be highly sensitive to high temperatures. Previous studies have shown that high temperatures severely reduce tomato plant net photosynthetic rate by decreasing tomato leaf pigment content, damaging leaf ultrastructure, and damaging plant Photosystem II [10,11]. Heat stress also impaired RuBisCo activase ability to maintain RuBisCo in an active form, which resulted in less carbon fixation [12]. The lower carbon fixation rate on the heat-stressed plant was also caused by reduced gas exchange due to stomatal closure [9,13]. Heat stress also uncouples enzymes and metabolic pathways, which cause the accumulation of reactive oxygen species (ROS) within the reaction centers of Photosystem I (PSI) and Photosystem II (PSII), peroxisomes, and mitochondria [10,14]. Accumulation of ROS within the aforementioned sites causes oxidative damage to plant photosynthetic machinery and reduces plant photosynthetic efficiency [9]. Maintaining normal photosynthetic capabilities during heat stress is one of the key signs of heat tolerance in tomatoes [12,15] of the most important phytohormones responsible for plant adaptation to various stresses, including heat stress, is auxin [16]. Auxin mediates stress response through a complex system of crosstalk between auxin, ethylene, and cytokinin [17]. Auxin also maintains ROS homeostasis, which acts as a stress detection tool for plants [18]. Whenever there are imbalances in ROS homeostasis caused by external factors such as heat stress or drought stress, auxin seeks to mediate it by increasing redox enzyme synthesis [17]. Thus, during stress, auxin levels within plant tissue would increase [16]. This increase in auxin levels would then be followed by the increased synthesis of enzyme and non-enzyme antioxidants [19]. Interestingly, increased levels of auxin-induced by either exogenous auxin applications or auxin overexpression mutants have been linked with increased activities of enzyme and non-enzyme antioxidants such as NADPH-thioredoxin reductase (NTRC), ascorbate peroxidase (APX), glutathione reductase (GR), catalase (CAT), superoxide dismutase (SOD), quinine reductase, peroxide dismutase, and proline [13,19,20,21,22,23,24,25].

Heightened auxin response and concentration within plant tissue have also been linked with better photosynthetic capabilities of *Brassica juncea* [13] and rice during heat stress [26]. Auxin prevented stomatal closure under heat stress conditions [13,26] and also prevented leaf cells and pigments from being damaged by ROS buildup [26]. Multiple studies aimed to study the effects of increased levels of endogenous auxin on plant tolerance to abiotic stresses have been conducted in potatoes [23], *Nicotiana tabacum* [24], and Arabidopsis [25], with positive results. However, the effects of heightened auxin levels on tomato photosynthetic capability under heat stress conditions still need to be well studied.

To study the effects of heightened auxin levels on tomato photosynthetic capabilities under heat stress conditions, two mutant Micro-Tom tomatoes—*iaa9-3* and *iaa9-5*—with elevated auxin response and synthesis, were selected for this study. *iaa9-3 and iaa9-5* mutant lines of tomatoes were generated by exposing Micro-Tom tomatoes to Ethyl methane sulfonate (EMS) [27]. Micro-Tom tomato was used to develop the mutants due to its advantages as a plant model. Micro-Tom tomato has a short lifespan (60–70 days), high population density, and the ability to produce fruit [28]. Increased auxin response and synthesis exhibited by both *IAA9* mutants were caused by the alteration of the *IAA9* gene [27]. The *IAA9* gene acts as a negative regulator of auxin [29]; hence the loss of function of *IAA9* will trigger increased auxin response [27]. This is confirmed by the finding of [30], which reported that upregulation of *SlIAA9* resulted in reduced auxin synthesis, while downregulation of *SlIAA9* resulted in increased auxin synthesis within plant tissue. It is also interesting to note that *iaa9-3* and *iaa9-5* have different degrees of heightened auxin response, with the latter exhibiting a stronger auxin response than the former [31]. In this paper, we studied the effects of different degrees of elevated auxin response and synthesis induced by *SlIAA9* mutation on tomato photosynthetic capability under heat stress conditions. We believed that heightened auxin response and synthesis caused by *SlIAA9* mutation would be beneficial in maintaining tomato photosynthesis capabilities under heat stress conditions by preventing leaf damage and maintaining leaf gas exchange capabilities.

## 2. Results

### 2.1. IAA9 Mutants Have Wider Individual Leaf Area and Heavier Leaf Weight under Heat Stress Conditions

**Figure 1:**
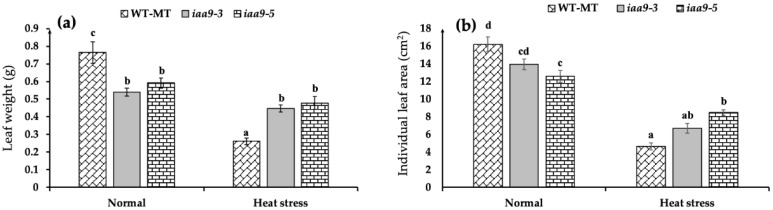
(**a**) Leaf weight of WT-MT, *iaa9-3*, and *iaa9-5* under normal and heat stress conditions; (**b**) individual leaf area of WT-MT, *iaa9-3*, and *iaa9-5* under normal and heat stress conditions. The average value ± standard error (SE) (*n* = 4) followed by the same letter is not significantly different according to the Tukey-HSD test at 5%.

The result of our study showed that *iaa9-3* and *iaa9-5* have lighter leaves than WT-MT under normal condition (Figure 1a). Under normal conditions, WT-MT has the heaviest individual leaf weight with an average weight of 0.765 g, followed by *iaa9-5* with an average individual leaf weight of 0.591 g, and *iaa9-3* with an average individual leaf weight of 0.54 g. The opposite is true under heat stress condition, *iaa9-3* and *iaa9-5* leaves were heavier than WT-MT (Figure 1a). Under heat stress condition, *iaa9-5* has the heaviest leaf weight with an average individual leaf weight of 0.478 g, followed by *iaa9-3* with an average individual leaf weight of 0.447 g, and finally WT-MT with an average individual leaf weight of 0.262 g (Figure 1a). Interestingly, heat stress severely reduced WT-MT leaf weight but not *iaa9-3* and *iaa9-5* mutants; heat stress reduced WT-MT individual leaf weight by 65.79%, while *iaa9-3* and *iaa9-5* weights were only reduced by 17.27% and 19.16%, respectively.

**Figure 2:**
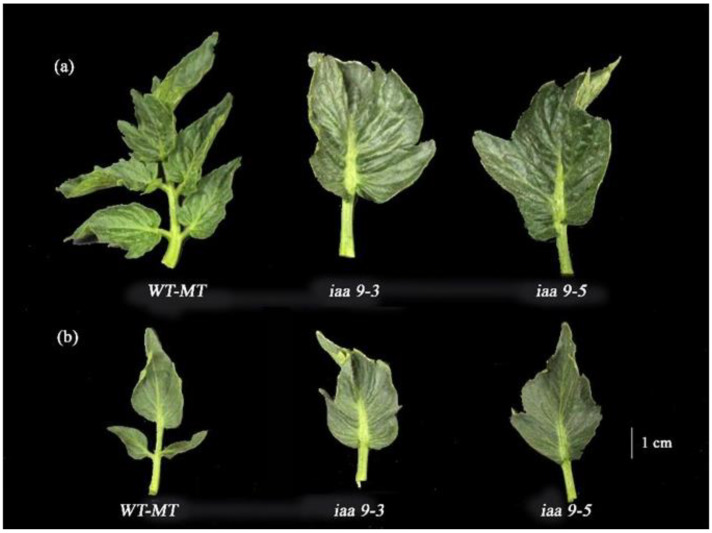
(**a**) WT-MT, *iaa9-3*, and *iaa9-5* leaf under normal conditions; (**b**) WT-MT, *iaa9-3*, and *iaa9-5* leaf under heat stress conditions.

Our observation shows that WT-MT has the widest individual leaf at normal temperature with an average value of 16.27 cm^2^. However, it was not statistically significant with *iaa9-3,* followed closely at 13.94 cm^2^, while *iaa9-5* has the narrowest individual leaf area at 12.58 cm^2^ (Figure 1b). Heat stress reduced all genotype individual leaf areas; under heat stress conditions, individual leaf areas became narrower (Figure 2). WT-MT was the most affected among all the genotypes; heat stress reduced WT-MT individual leaf area by 71.4%, while *iaa9-3* and *iaa9-5* individual leaf areas were reduced by 52% and 32.63%, respectively. The average individual leaf area of WT-MT, *iaa9-3*, and *iaa9-5* under heat stress conditions were 4.64, 6.69, and 8.475 cm^2^, respectively.

### 2.2. IAA9 Mutants Have Higher Net Photosynthetic Rate, Stomatal Conductance, and Water Use Efficiency under Heat Stress Conditions

**Figure 3:**
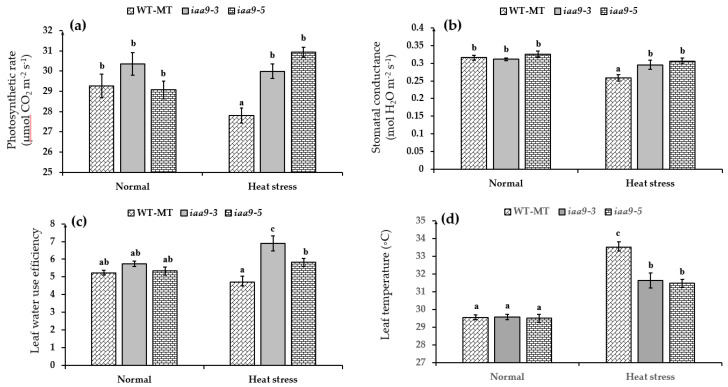
(**a**) Plant photosynthetic rate of WT-MT, *iaa9-3*, and *iaa9-5* under normal and heat stress conditions; (**b**) leaf stomatal conductance of WT-MT, *iaa9-3*, and *iaa9-5* under normal and heat stress conditions. (**c**) Water use efficiency of WT-MT, *iaa9-3*, and *iaa9-5* under normal and heat stress conditions. (**d**) Leaf temperature of WT-MT, *iaa9-3*, and *iaa9-5* under normal and heat stress conditions. The average value ± standard error (SE) (*n* = 4) followed by the same letter is not significantly different according to the Tukey-HSD test at 5%.

Plant photosynthesis is known to be sensitive to high temperatures. Interestingly, *iaa9-3* and *iaa9-5* mutants were seemingly unaffected by heat stress conditions and managed to maintain a similar net photosynthetic rate to normal conditions. Under normal temperatures, *iaa9-3,* and *iaa9-5* net photosynthetic rates were 30.36 and 29.07 μmol CO_2_ m^−2^·s^−1^, respectively (Figure 3a), while under heat stress conditions, *iaa9-3* and *iaa9-5* net photosynthetic rate were 29.99 and 30.94 μmol CO_2_ m^−2^·s^−1^, respectively. WT-MT net photosynthetic rate under heat stress significantly declined by 1.45 μmol CO_2_ m^−2^·s^−1^ compared with normal conditions (Figure 3a). WT-MT net photosynthetic rates were 29.26 and 27.81 μmol CO_2_ m^−2^·s^−1^ under normal and heat stress conditions, respectively (Figure 3a). This study observed leaf stomatal conductance as an effect of *IAA9* mutation under normal and heat stress conditions. Compared to WT-MT, the result showed a significant impact of IAA9 mutation in stomatal conductance under heat stress conditions. Under heat stress conditions, *iaa9-3* and *iaa9-5* mutants maintained higher stomatal conductance than WT-MT and were not significantly different compared to under normal conditions (Figure 3b). Under normal temperature WT-MT, *iaa9-3* and *iaa9-5* have similar stomatal conductance values at 0.315, 0.31, and 0.326 mmol H_2_O m^−2^·s^−1^, respectively. A significant decrease in stomatal conductance under heat stress conditions was detected in WT-MT, while in *iaa9-3* and *iaa9-5,* it was not detected. Under heat stress conditions, the values of stomatal conductance were 0.258, 0.295, and 0.305 mmol H_2_O m^−2^·s^−1^, respectively, for WT-MT, *iaa9-3*, and *iaa9-5* (Figure 3b).

Leaf water use efficiency (WUE) of investigated leaves varied when the plants were treated with heat stress, but there was similar under normal conditions. The value WT-MT leaf WUE under heat stress conditions decreased by 0.50 from normal conditions or 5.21 to 4.71, but it was not statistically significant between normal and heat stress conditions (Figure 1c). On the other hand, the *iaa9-3* mutant significantly increased leaf WUE under heat stress conditions compared to normal conditions. Meanwhile, *iaa9-5* leaf WUE under heat stress conditions was not significantly different from normal conditions (Figure 3c). Under normal conditions, *iaa9-3* and *iaa9-5* leaf WUE were 5.73 and 5.33, respectively; under heat stress conditions, they were 6.89 and 5.81, respectively. This result suggests that *SlIAA9* mutation could increase leaf WUE under heat stress conditions.

The leaf temperature of each tomato plant was similar under normal conditions. Under normal conditions, the average leaf temperature of WT-MT, *iaa9-3*, and *iaa9-5* was 29.53, 29.56, and 29.49 °C*,* respectively. Heat stress conditions increased each genotype’s leaf temperatures. However, WT-MT leaf temperature was the most affected by heat stress conditions. Under heat stress conditions, WT-MT leaf temperature rose by 3.971 °C. In contrast, *iaa9-3* and *iaa9-5* were less affected by heat stress conditions. Under heat stress conditions, *iaa9-3* and *iaa9-5* leaf temperatures rose by 2.068 and 1.98, respectively (Figure 3d). This result suggests that the *Sliaa9* mutation increased leaf cooling ability under heat-stress conditions.

### 2.3. IAA9 Mutants Have Higher Leaf Chlorophyll and Carotenoid Content

**Figure 4:**
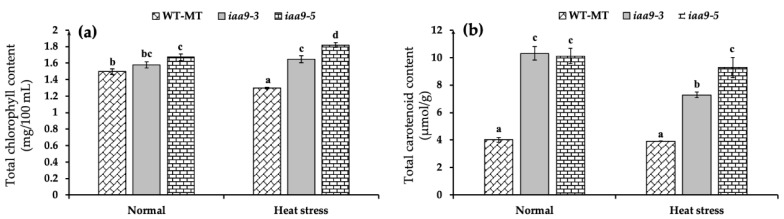
(**a**) Leaf total chlorophyll content of WT-MT, *iaa9-3*, and *iaa9-5* under normal and heat stress conditions; (**b**) leaf total carotenoid content of WT-MT, *iaa9-3*, and *iaa9-5* under normal condition and heat stress conditions. The average value ± standard error (SE) (*n* = 4) followed by the same letter is not significantly different according to the Tukey-HSD test at 5%.

Based on our observation, under normal conditions, *iaa9-5* mutant tomato has the highest leaf chlorophyll content and significantly higher than WT-MT with an average value of 1.67 mg/100 mL and 1.49 mg/100 mL, respectively, for *iaa9-3* and W-MT. However, it was not significantly different from WT-MT for *iaa9-3,* with an average value of 1.57 mg/100 mL (Figure 4a). Heat stress treatment significantly reduced the leaf chlorophyll content of WT-MT by 13.31% lower than under normal conditions. Interestingly, the leaf chlorophyll content of mutants was less affected by heat stress; the *iaa9-5* leaf chlorophyll content even significantly increased under heat stress compared with normal conditions (Figure 4a).

Mutation in the *SlIAA9* gene significantly affects the increasing total leaf carotenoid content, proven by significantly higher leaf carotenoid content of *iaa9-3* and *iaa9-5* mutants in either normal or heat stress conditions (Figure 4b). Under normal conditions, the leaf carotenoid contents of *iaa9-*3 and *iaa9-5 were* 157.5 and 152.5%, higher than WT-MT, which has the leaf carotenoid content of 4 μmol/g. Heat stress decreased the carotenoid content of all genotypes, but the decrease was not statistically significant for *iaa9-5* and WT-MT. Under heat stress conditions, WT-MT, *iaa9-*3, and *iaa9-5* leaf carotenoid content were reduced to 3.91, 7.28, and 9.26 μmol/g, respectively. Interestingly, the *iaa9-3* leaf carotenoid seemed more affected by heat stress than both *iaa9-5* and WT-MT (Figure 4b).

### 2.4. IAA9 Mutants Have Lower Leaf Malondialdehyde (MDA) Content during Heat Stress

**Figure 5:**
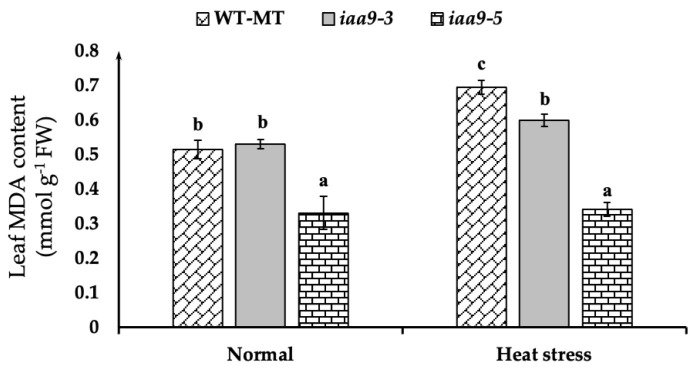
Leaf MDA content of WT-MT, *iaa9-3*, and *iaa9-5* under normal and heat stress conditions. The average value ± standard error (SE) (*n* = 4) followed by the same letter is not significantly different according to the Tukey-HSD test at 5%.

Malondialdehyde (MDA) was used as an indicator to measure plant tissue damage. We observed that *iaa9-5* has the lowest leaf MDA content at both temperatures (Figure 5). At normal temperature, *iaa9-5* has an average MDA content of 0.33 mmol·g^−1^ FW, which is significantly lower than both *iaa9-3* and WT-MT, with the value of 0.53 and 0.51 mmol·g^−1^ FW, respectively. At high temperatures, the leaf MDA content of each genotype was varied. WT-MT has a significant increase in MDA compared to normal conditions and resulted in the highest leaf MDA content with the value of 0.7 mmol·g^−1^ FW or 0.17 higher than normal conditions. Interestingly, for *iaa9-3* and *iaa9-5*, heat treatment did not affect the increasing MDA that was statistically comparable with the MDA under normal conditions. The MDA of *iaa9-3* and *iaa9-5* under heat stress conditions were 0.59 and 0.34 mmol·g^−1^ FW, respectively, which have increased 0.07, and 0.01 mmol·g^−1^ FW, respectively, compared to normal conditions (Figure 5).

### 2.5. IAA9 Mutants Shown Better Chlorophyll Fluorescence under Heat Stress Conditions

**Figure 6:**
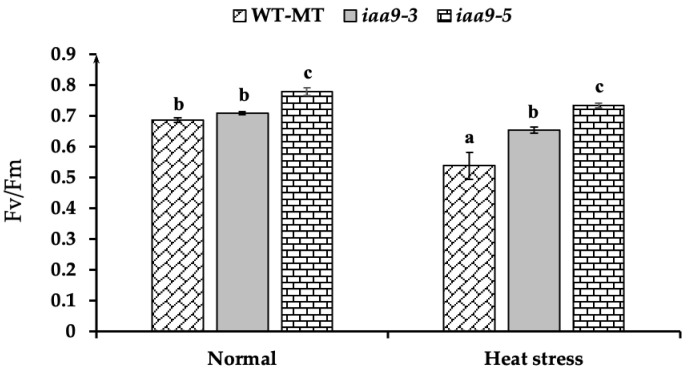
Chlorophyll fluorescence (Fv/Fm) value of WT-MT, *iaa9-3*, and *iaa9-5* under normal and heat stress conditions. The average value ± standard error (SE) (*n* = 4) followed by the same letter is not significantly different according to the Tukey-HSD test at 5%.

Chlorophyll fluorescence (Fv/Fm) was measured to assess the degree of stress-induced damage to photosynthetic structures. In this study, *iaa9-5* has the highest chlorophyll fluorescence value under normal conditions at 0.78, significantly higher than WT-MT at 0.68 and *iaa9-3* at 0.71 (Figure 6). After 14 days of heat treatment, a reduction in chlorophyll fluorescence was observed in WT-MT and *iaa9-5*, whereas it was not affected in *iaa9-3* (Figure 6). Under heat stress conditions, both *iaa9-3* and *iaa9-5* have higher chlorophyll fluorescence values than WT-MT, with the chlorophyll fluorescence value of WT-MT, *iaa9-*3, and *iaa9-5* being 0.53, 0.65, and 0.73, respectively (Figure 6). Higher chlorophyll fluorescence of both *iaa9-3* and *iaa9-5* can be attributed to lower MDA content, indicating less leaf oxidative damage.

### 2.6. Multivariate Analysis of Analyzed Trait under Heat Stress Conditions

Pearson correlation analysis of the analyzed trait under heat stress conditions yielded 17 strong positive correlations between each analyzed trait (coef. > 0.7) and 12 negative strong correlations between each analyzed trait (coef. < −0.7). Traits that have a strong positive correlation to plant net photosynthetic rate (Pn) during heat stress were leaf total chlorophyll content (r 0.88), leaf total carotenoid content (r 0.77), Fv/Fm value (r 0.78), leaf weight (r 0.93), and leaf area (r 0.92), while stomatal conductance (r 0.58) and leaf water use efficiency (0.59) have a moderate positive correlation with Pn during heat stress. On the other hand, traits that have a strong negative correlation with Pn during heat stress were leaf MDA content (r −0.87) and leaf temperature (r −0.89) (Table 1). This result suggests that Pn during heat stress was most affected by changes in the following traits: leaf total chlorophyll content, leaf total carotenoid content, leaf MDA content, Fv/Fm value, leaf area, leaf temperature and leaf weight, while changes in stomatal conductance and leaf WUE have less impact on Pn during heat stress conditions.

## 3. Discussion

Several studies have highlighted the importance of studying plant leaves as a proxy for plant growth and plant physiology [32,33,34,35,36]. Leaf size and weight are important morphological characteristics often observed to measure plant ability to accumulate biomass [37,38,39,40]. WT-MT leaves were broader and heavier than *iaa9-5* under normal conditions (Figure 1a,b). This is likely due to *SlIAA9* mutation, which caused morphological changes in Micro-Tom tomato (Figure 2); iaa9-3 and *iaa9-5* leaves were not trifoliate, differing from their WT-MT counterpart leading to a more compact leaf structure [41]. This finding also suggests that auxin controls the expression of the *Trifoliate* (*Tf*) gene, the gene responsible for inducing leaf trifoliate growth in tomatoes [42]. Interestingly, both *iaa9-3* and *iaa9-5* mutants leaf weight and individual leaf area were not affected by heat stress, thus differing from WT-MT (Figure 1a,b). This suggests that the mutants mentioned above could accumulate biomass even under heat stress conditions, which was proven by their higher net photosynthetic rate under heat stress conditions (Figure 3a).

Photosynthesis is known to be very sensitive to temperature [9,12]. Therefore, maintaining a normal net photosynthesis rate at high temperatures is a sign of heat tolerance [9,13,43]. In this study, we discovered that WT-MT has a similar net photosynthetic rate with *iaa9-3* and *iaa9-5* under normal conditions; however, under heat stress conditions, WT-MT net photosynthetic rate was lower than *iaa9-3* and *iaa9-5* mutants (Figure 3a). This was likely caused by lower stomatal conductance (Figure 3b), lower leaf pigment contents of WT-MT tomato under heat stress conditions (Figure 4a,b), and also higher rate of photosynthetic apparatus damages indicated by higher MDA content and low chlorophyll fluorescence (Figure 5 and Figure 6). On the other hand, higher net photosynthetic rate (Pn) observed in *iaa9-3* and *iaa9-5* under heat stress conditions were likely caused by better leaf evaporative cooling (Figure 3d), stomatal conductance (Figure 3b), higher leaf pigment contents (Figure 4a,b), lower photosynthetic apparatus damages indicated by lower MDA content (Figure 5), and higher Fv/Fm content (Figure 6). The aforementioned traits are vital in maintaining good photosynthetic capabilities under heat stress conditions, as shown in Table 1. This result suggests that *IAA9* mutation enhances tomato tolerance to heat by maintaining its photosynthetic capabilities under heat stress conditions.

Stomata facilitate gas and water exchange between plants and the surrounding environment [44], and lower stomatal conductance was often linked with a lower net photosynthetic rate [45]. CO_2_ used for photosynthesis was acquired through the stomata opening [46]. Under heat stress conditions, opened stomata also allowed for evaporative cooling of the leaves [47]. This evaporative cooling is vital since, at high leaf temperature, RuBisCo activase’s ability to maintain RuBisCo in an active form is impaired, which results in less carbon fixation [12]. Thus, tomatoes’ ability to prevent stomatal closure during heat stress has been attributed to heat tolerance [46]. Higher stomatal conductance observed on *iaa9-3* and *iaa9-5* was likely caused by *Sliaa9* mutation. Previous research has linked increased auxin content within the leaves with higher stomatal conductance on normal [13] and heat stress conditions [48]. Auxin helps prevent stomatal closure by increasing antioxidants within the leaves [17], thus mediating ROS accumulation at stomatal guard cells [13]; auxin also prevents stomatal closure by increasing leaf ethylene content which inhibits ABA synthesis in the leaf [48].

Based on our observation, *iaa9-3* has the highest leaf WUE of all experimental plants under heat stress conditions (Figure 3c). This finding suggested that *iaa9-3* was more efficient in preserving and utilizing water for photosynthesis. Previous research has shown that auxin application increased plant WUE; however, the exact mechanism was still unclear [49].

Our study also revealed that *iaa9-3* and *iaa9-5* have lower leaf temperatures compared to WT-MT under heat stress conditions (Figure 3d). This was likely caused by higher stomatal conductance of both mutants during the study (Figure 3b). Opened stomata under heat stress conditions allowed for evaporative cooling of the leaves to take place, thus reducing leaf overall temperature [39].

During heat stress, leaf chlorophyll content is a major factor in determining plant photosynthetic capability because in the absence of irreversible injury to the photosystem, high chlorophyll content allows the plant to restore leaf photo assimilation once stomatal conductance increases [46]. Based on our observation, under heat stress conditions, WT-MT leaf total chlorophyll content was significantly reduced (Figure 4a). This was likely caused by chlorophyll degradation [39] and impaired 5-aminolevulinic acid and protochlorophyllide biosynthesis, which are vital for chlorophyll biosynthesis [12]. WT-MT leaf carotenoid content was not affected by heat stress. This is because carotenoids are less temperature sensitive [50]. Interestingly, *iaa9-5* mutants have higher total leaf chlorophyll and carotenoid contents than WT-MT under normal and heat stress conditions (Figure 4a,b). Previous research has shown that increased auxin level within plant tissue enhances chlorophyll accumulation [51], however, the exact mechanism of how auxin affects chlorophyll biosynthesis pathways is still largely unknown [52]. On the other hand, increased leaf carotenoid contents of *iaa9-3* and *iaa9-5* under normal conditions might be an antioxidant response to oxidative stress present in both *IAA9* mutants even under normal conditions [46] since increased auxin concentration within plant tissue has been linked with increased ROS within plant tissue [53]. Higher leaf carotenoids of both *iaa9* mutants were beneficial for their photosynthetic capabilities since carotenoids were known to mediate photo-oxidative stress and also help in heat dissipation [54]. Carotenoids are also known for their importance as a light-harvesting apparatus during environmental stress [55]. Higher levels of chlorophyll and carotenoid contents of *iaa9-3* and *iaa9-5* leaves under heat stress conditions were likely caused by lower leaf surface temperature due to better evaporative cooling indicated by higher stomatal conductance (Figure 3b) and a higher level of antioxidants which prevented tissue damages indicated by lower leaf MDA content (Figure 5).

Malondialdehyde (MDA) is a good indicator of lipid peroxidation [56]. Thus, it is often used as an indicator to measure damage to plant tissues [57]. Previous studies have shown that lower MDA content was a sign of a heat-tolerant tomato [11,15,57]. Both *iaa9-3* and *iaa9-5* have lower leaf MDA content than WT-MT under heat stress conditions, especially *iaa9-5*. A similar result was reported by [26], auxin helped prevent leaf and photosynthetic apparatus damage under stress conditions. This is because auxin maintains ROS homeostasis within plant tissue, auxin mediates ROS imbalances caused by abiotic stresses within plant tissue by increasing redox enzyme synthesis, thus preventing tissue damage. Previous research has established a link between higher auxin levels within plant tissue and higher SOD, CAT, APX, Glutathione, and NADPC activities [13,20,21,22,23,24,25]. *iaa9-3* and *iaa9-5* were also known to have higher proline content under simulated drought stress [20]. The aforementioned antioxidants are important ROS scavenger enzymes [18]. Higher leaf carotenoid content of *iaa9-3* and *iaa9-5* may also help prevent photosystem damage (Figure 4b) since carotenoids were known to mediate photo-oxidative stress and also helps in heat dissipation [54]. This result suggests that heightened auxin response and biosynthesis due to *IAA9* mutation were beneficial for preventing leaf tissue damage (Figure 5).

Chlorophyll fluorescence (Fv/Fm) measurement helps to assess the degree of stress-induced damage to photosynthetic structures [47]. A lower chlorophyll fluorescence value indicates that plant photosynthetic apparatus was damaged by oxidative stress [15], while a high chlorophyll fluorescence value indicates tolerance to heat stress [9]. WT-MT has the lowest Fv/Fm value amongst the experimental plants (Figure 6), with an average value of 0.53, suggesting substantial damage to PSII and indicating the onset of severe stress [46]. In contrast, both *iaa9-3* and *iaa9-5* mutants have higher chlorophyll fluorescence under normal and heat stress conditions (Figure 6). Higher chlorophyll fluorescence of both *iaa9-3* and *iaa9-5* can be attributed to lower MDA content (Figure 5), which indicates less leaf oxidative damages [44], higher leaf chlorophyll and carotenoid contents, which helps prevent photo-oxidative stress [55], and higher stomatal conductance which prevents Fq/Fm reduction [46].

## 4. Materials and Methods

### 4.1. Growth Condition and Plant Material

The experiment was conducted inside a greenhouse at Universitas Padjadjaran, Indonesia, where the average temperature (27 ± 3 °C) and relative humidity (35–40%) was maintained for normal condition. The average maximum daily temperature for normal conditions during the study was 33.05 °C, while the average minimum daily temperature was 21.32 °C. Following the methods described by [58], we made a heat stress chamber within the same greenhouse where the experiment was conducted, the average temperature inside the heating chamber is 40 ± 3 °C, and its average relative humidity is 50–60%. The average maximum daily temperature for normal conditions during the study was 42.84 °C, while the average minimum daily temperature was 24.57 °C. The heat treatment was conducted for 14 days. We recorded the temperature and relative humidity inside the greenhouse and the heating chamber using an HTC-1 Digital Thermo-Hygrometer (HTC Instruments, Mumbai, India).

Wild-Type Micro-Tom tomato (*Solanum lycopersicum*)/WT-MT, *iaa9-3*, and *iaa9-5* Micro-Tom tomato were obtained from the University of Tsukuba, Japan. The *iaa9-3* and *iaa9-5* mutants were created through mutation breeding using EMS [27]. The seeds were germinated in a seed tray filled with a mixture of cocopeat and charcoal husk (1:1/*v:v*). After 14 days, the seedlings were transplanted into 12 cm pots filled with cocopeat and charcoal husk (1:1/*v:v*). After transplantation, throughout the experiment, pots were watered and fertilized daily using AB Mix (2500 ppm, 2.2 EC, pH 6). Plants were exposed to heat stress two weeks after transplanting. A simple randomized design was chosen with four batches, each containing four plants for each treatment. Then WT-MT, *iaa9-3,* and *iaa9-5 were* grown under two conditions: normal and heat stress.

### 4.2. Individual Leaf Area and Leaf Weight

Plant leaf area was measured using ImageJ, following the methods used by [59]. Briefly, a single leaf tomato leaf was washed and laid down on a white sheet of paper; pictures were then taken using the same angle and distance from the observed leaves. The photos were then analyzed using ImageJ. While leaf weight was measured utilizing an analytical balance.

### 4.3. Plant Photosynthetic Rate, Stomatal Conductance, and Leaf Temperature

Plant photosynthetic rate and stomatal conductance were measured using a Li-6400 XT (Licor Inc., Lincoln, NE, USA). Measurement was conducted on a sunny day at around 9.30 a.m. WIB (GMT + 7). Only pest and disease-free third leaves were used as samples, and each sample leaf was also measured three times. Heat-stressed leaves were measured under heat-stress conditions.

### 4.4. Leaf Water Use Efficiency

Leaf water use efficiency (WUE) was determined utilizing data given by Li-6400 XT (Licor Inc., Lincoln, NE, USA). Leaf WUE measurement was conducted on a sunny day at around 9.30 a.m. WIB (GMT + 7). Pest- and disease-free third leaves were chosen as samples, and each sample leaf was measured three times. Heat-stressed leaves were measured under heat-stress conditions.

### 4.5. Total Leaf Chlorophyll and Total Carotenoid Content

Total leaf chlorophyll and carotenoid content were measured following the methods described by [60]. One gram of fresh leaf sample was ground and combined with 10 mL of 80% acetone until the leaf sample was whitened. The extract was then filtrated using a Whatman paper into a cuvette to be analyzed using a spectrophotometer (Orion™ AquaMate 8000, Thermo Fisher Scientific, Horsham, UK) at 480, 645, and 663 nm. Leaf chlorophyll and carotenoid content were then measured using the following equations:

Total chlorophyll content mg/L = 8.02 × A663 + 20.2 × A645

$$\mathrm{Total carotenoid content}\;\mu \mathrm{mol/g}\;=\;\frac{(A_{480}\;+\;0.114\;\times \;A_{663}\;-\;0.638\;\times \;A_{645})\;\times \;V\;\times \;10^{3}}{112.5\;\times \;W}$$

V = Volume of mixture in L

W = Weight of leaf sample in g

### 4.6. Leaf MDA Content

Leaf MDA content was measured following the methods used by [57]. A total of 0.3 g of leaf fresh sample was combined with 3 mL of 10% (*m/v*) trichloroacetic acid (TCA) and then centrifuged at 10,000 rpm for 15 min. Afterward, 2 mL of supernatant was combined with 2 mL of 0.6% thiobarbituric acid (*w/v*). The mixture was heated in boiling water for 20 min and then cooled to room temperature. Then the mixture was centrifuged again at 10,000 rpm for 15 min. After centrifugation, the supernatant absorbance was measured using a spectrophotometer (Orion™ AquaMate 8000, Thermo Fisher Scientific, UK) at 450, 532, and 600 nm. The sample MDA content can then be measured using the following formula:

C_MDA_ (μmol·L^−1^) = 6.45*(A_532_ − A_600_) − 0.56*A_450_.

C_MDA_ = Leaf total MDA content

### 4.7. Chlorophyll a Fluorescence

Chlorophyll fluorescence was measured using a Handy PEA fluorometer (Hansatech Instruments Ltd., Narborough, UK), following the methods described by [11]. Briefly, leaves were dark adapted for 30 min before measurement using a Handy PEA fluorometer.

## 5. Conclusions

This study observed the photosynthetic capability of *IAA9* Micro-Tom tomato mutants, *iaa9-3* and *iaa9-5*, with different levels of heightened auxin response under heat stress conditions compared to WT-MT tomato. *SlIAA9* mutation increased tomato leaf evaporative cooling capabilities by preventing stomatal closure, thus allowing for leaf gas exchange and carbon fixation under heat stress conditions. Both *iaa9* mutants also have less ROS accumulation within their leaves, indicated by lower MDA content and higher Fv/Fm value. We believed this was likely caused by lower leaf temperature and by increased antioxidant and non-antioxidant enzyme synthesis within *iaa9-3* and *iaa9-5* mutants. Thus, further research should focus on phytochemical changes induced by the *SlIAA9*mutation

## Figures and Tables

**Table 1:** Pearson correlation between analyzed traits under heat stress conditions.

	CHL	CR	MDA	CI	SC	WUE	LT	PN	LW	LA
CHL	0.97									
CR	0.86	0.97								
MDA	−0.89	−0.78	0.98							
CI	0.89	0.71	−0.84	0.99						
SC	0.65	0.70	−0.51	0.47	1.00					
WUE	0.64	0.38	−0.31	0.52	0.36	0.97				
LT	−0.93	−0.81	0.74	−0.80	−0.68	−0.69	0.97			
PN	0.88	0.77	−0.87	0.78	0.58	0.59	−0.89	0.98		
LW	0.92	0.80	−0.74	0.85	0.66	0.65	−0.97	0.93	0.94	
LA	0.88	0.84	−0.89	0.79	0.54	0.40	−0.87	0.92	0.79	0.97

Note: CHL-leaf total chlorophyll content, CR-leaf total carotenoid content, MDA-leaf total MDA content, CI-Fv/Fm value, SC-stomatal conductance, WUE-leaf water use efficiency, PN-net photosynthetic rate, LW-leaf weight, LA-leaf area. Deep green-strong correlation, green-medium correlation, light green-weak correlation, deep grey-weak negative correlation, light grey-medium negative correlation, white-strong negative correlation.

## Data Availability

Not applicable.
